# Developmental expression and differentiation-related neuron-specific splicing of *metastasis suppressor 1 *(Mtss1) in normal and transformed cerebellar cells

**DOI:** 10.1186/1471-213X-7-111

**Published:** 2007-10-09

**Authors:** Alexander Glassmann, Sabine Molly, Lachezar Surchev, Tommy A Nazwar, Martin Holst, Wolfgang Hartmann, Stephan L Baader, John Oberdick, Torsten Pietsch, Karl Schilling

**Affiliations:** 1 Anatomisches Institut, Anatomie & Zellbiologie, University of Bonn, Bonn, Germany; 2 Institut für Neuropathologie, University of Bonn, Bonn, Germany; 3 Institut für Anatomie, University of Jena, Jena, Germany; 4 Center for Molecular Neurobiology, Department of Neuroscience, The Ohio State University, Columbus, OH, USA; 5 Department of Anatomy, Histology and Embryology, Medical University Sofia, BG-1431 Sofia, Bulgaria

## Abstract

**Background:**

Mtss1 encodes an actin-binding protein, dysregulated in a variety of tumors, that interacts with sonic hedgehog/Gli signaling in epidermal cells. Given the prime importance of this pathway for cerebellar development and tumorigenesis, we assessed expression of Mtss1 in the developing murine cerebellum and human medulloblastoma specimens.

**Results:**

During development, Mtss1 is transiently expressed in granule cells, from the time point they cease to proliferate to their synaptic integration. It is also expressed by granule cell precursor-derived medulloblastomas. In the adult CNS, Mtss1 is found exclusively in cerebellar Purkinje cells. Neuronal differentiation is accompanied by a switch in Mtss1 splicing. Whereas immature granule cells express a Mtss1 variant observed also in peripheral tissues and comprising exon 12, this exon is replaced by a CNS-specific exon, 12a, in more mature granule cells and in adult Purkinje cells. Bioinformatic analysis of Mtss1 suggests that differential exon usage may affect interaction with Fyn and Src, two tyrosine kinases previously recognized as critical for cerebellar cell migration and histogenesis. Further, this approach led to the identification of two evolutionary conserved nuclear localization sequences. These overlap with the actin filament binding site of Mtss1, and one also harbors a potential PKA and PKC phosphorylation site.

**Conclusion:**

Both the pattern of expression and splicing of Mtss1 is developmentally regulated in the murine cerebellum. These findings are discussed with a view on the potential role of Mtss1 for cytoskeletal dynamics in developing and mature cerebellar neurons.

## Background

A fundamental aspect of nervous system development and function is the expansion of basic cellular polarity into a highly diverse array of neural phenotypes specialized for directional information processing. Morphological polarization of glia and neurons alike has long been used as a basic principle for classification of these cells, which was fundamental to the initial understanding of their functions and interactions [1]. In neurons, the formation and maintenance of dendrites and axons clearly is one of the most palpable aspects of developmental polarization. Function-driven plasticity, and post-traumatic reaction of neuronal processes, document that polarization is dynamic and needs to be regulated beyond the stage of development proper; in addition, these phenomena point to the potential practical clinical significance of understanding basic mechanisms of neural polarization from molecular principles.

The cytoskeleton is center stage to the mechanistic realization of cellular polarity (e.g., [2-4]. It provides a scaffold to transduce reactions to external signals, and also to link and subcellularly segregate molecular constituents of the signal transduction machinery. Molecularly, these functions are implemented through a large array of proteins, referred to as cytoskeleton-associated, or -binding, proteins (for reviews, see e.g. [5,6]). In neurons, the differential subcellular distribution of distinct subsets of these proteins has been related to neurite identity, cytoskeletal organization, and function (e.g. [4,7-9]).

Mtss1 (metastasis suppressor 1; also known as missing in metastasis, MIM, or BEG4, and initially isolated as KIAA0429; cf [10,11]) is a recently identified actin-binding protein. It has been implicated in the regulation of actin filament assembly [12-15], in mediating interaction of the cytoskeleton with phosphatidylinositol 4,5-bisphosphate-rich membranes, and membrane bending [16,17]. Mtss1 is expressed in several embryonic tissues including the developing central nervous system. In the adult central nervous system, it is prominently expressed by cerebellar Purkinje cells [12]. It has also been observed to be down-regulated, or missing, in several metastatic cancer cell lines [11,18,19].

In the epidermis, Mtss1 has been identified as a Sonic Hedgehog (Shh)-responsive gene that modulates Gli-regulated transcription [20]. As multiple members of the Shh-Gli pathway are strongly expressed in the cerebellar anlage during the key phase of neural migration and morphogenesis in this structure (e.g., [21-23]), and indeed are necessary for the orderly development of the cerebellum, we sought to elucidate the expression of Mtss1 in the developing and adult cerebellum and relate it to that of members of the Shh-signaling pathway on the one hand, and defined steps of cerebellar morphogenesis on the other.

Our results document a transient developmental expression of Mtss1 in granule cells which closely parallels their migration and neuritogenesis. Neuronal maturation is accompanied by a switch in splice variant expression of Mtss1. Bioinformatic analysis of the resulting protein isoforms reveals that they may differentially interact with several proteins previously identified as critical for cerebellar development and function and suggests that continued expression of Mtss1 in adult Purkinje cells relates to their functional plasticity.

## Results

### Expression of Mtss1 in the developing cerebellum

**Figure 1:**
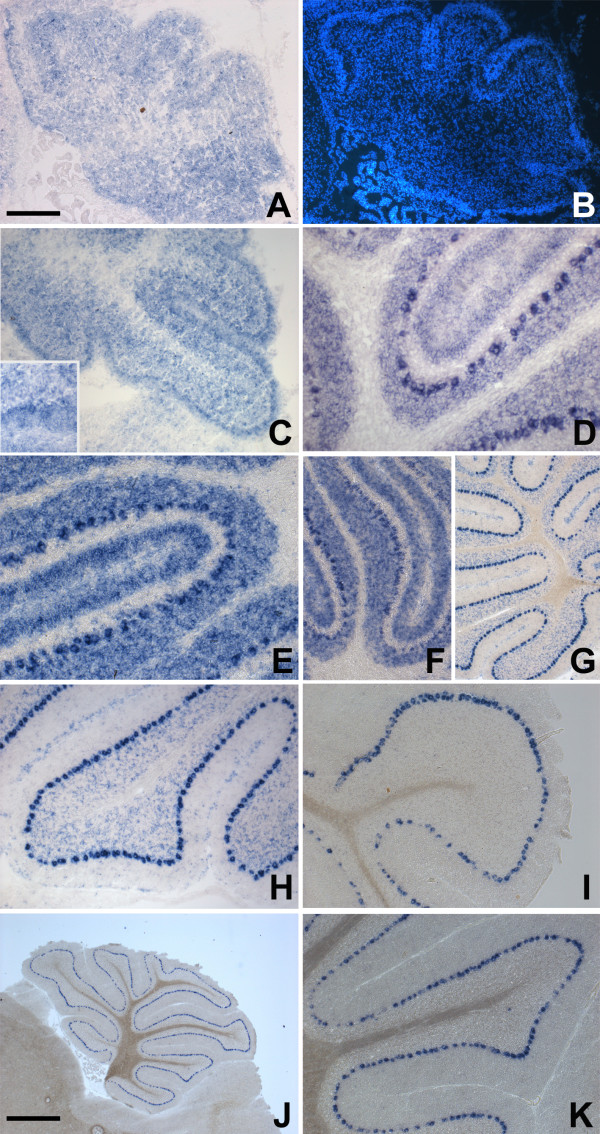
**Localization of Mtss1 mRNA in the developing and adult cerebellum**. **A, B**: In the newborn cerebellum, Mtss1 is found in a broad band of cells roughly outlining the nascent cerebellar cortex. Note that the deep cerebellar mass is relatively devoid of signal, as is the external granule cell layer. The latter can be readily recognized as a dense band at the surface in panel B, which shows counterstaining with Hoechst 33342. **C-F**: At p3 (C), p5 (D) and p8 (E, F), staining can be unambiguously attributed to cells in the inner part of the external granule cell layer, granule cells in the inner granule cell layer, and Purkinje neurons, which show an increasingly strong signal in the perikaryon. Note that the outer part of the EGL and the (prospective) white matter are devoid of signal. **G-I**: Between postnatal day 15 (G, H) and 21 (I), granule cells in the (internal) granule cell layer cease to express Mtss1. **J, K**: Adult. Staining is limited to Purkinje cell perikarya. All sections were obtained from the central vermis, except the one shown in panel I, which originates from the lateral vermis, and are cut in the sagittal plane. Anterior is to the left. Bar (in A, J) = 125 μm for panels D, E and insert in C; 250 μm for panels A, B, C, F, H and I; 500 μm for panels G and K; and 1 mm for panel J.

**Figure 2:**
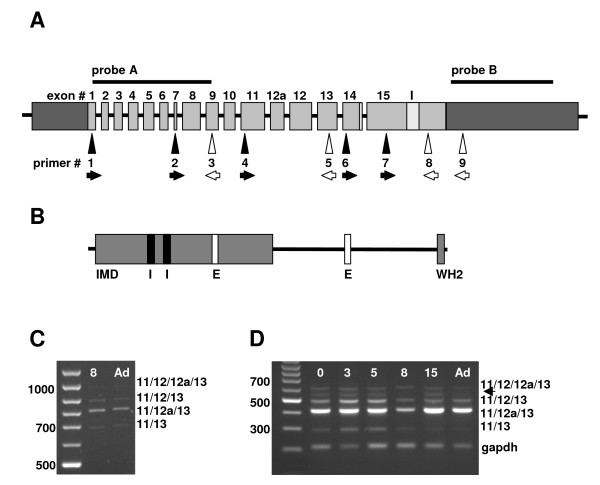
**A: **Schematic view of the organization of the murine Mtss1 gene based on Ensemble entry ENSMUSG00000022353. The 5' and 3' UTRs (dark boxes) are not drawn to scale, nor are the intronic regions. Comparison of the murine gene with that of the rat (ENSRNOG00000009001; transcript ENSRNOT00000023505) further suggests that the region labeled here as exon 15 may contain an additional, 108 bp long intron (I, marked in light gray) which would result in the division of exon 15 into two exons of 354 and 239 bps, respectively. Also, the length of exon 14 may either encompass 163 (ENSMUST00000080371) or 208 bp (ENSMUST00000036782). The 45 bps in question are located C-terminal and alternatively form part of the intron separating exons 14 and 15. Regions covered by the in situ hybridization probes used are labeled by horizontal lines A and B. Arrows mark positions of primers used (black arrows, forward primers; open arrows, reverse primers). **B: **Schematic view of the derived protein. The N-terminal IMD domain and the C-terminal WH2 domain are shown as gray boxes. The localization of the putative nuclear import (I) and export (E) motives are indicated as black and white boxes, respectively. **C, D: **Expression of Mtss1 splice variants in the early postnatal and adult murine cerebellum. Use of primers located in exons 7 and 13 (primers 2, 5; panel C) reveals the existence of 4 splice variants (the band representing exon combination 11/12/12a/13 reproduces only very weakly here) in the developing and adult cerebellum, as does the use of primers located in exons 11 and 13 (primers 4, 5; panel D). Note that the relative intensity in particular of the band representing splice variants comprising exons 11/12/13 and 11/12a/13 varies during development. The band labeled gapdh is a loading control. Numbers indicate days postnatal; Ad, adult. The arrow indicates a spurious amplificate from an unrelated sequence: This was verified by sequencing, as were the products labeled as bands 11/12/12a/13, 11/12/13, 11/12a/13 and 11/13.

Starting from the observation of Mattila et al [12], who documented expression of Mtss1 in adult Purkinje cells, we scrutinized Mtss1 expression in the developing cerebellum, from the day of birth into adulthood. In newborn (p0) animals, labeled cells were arranged in a broad band which outlined the incipient cerebellar folia. Both the deep cerebellar mass and the external granule cell layer were labeled only weakly, if at all (Fig 1A, B). At postnatal day 3 (p3), when individual cerebellar cortical layers could be better told apart, a clear label was detected over the Purkinje cell layer (which, as typical for this age, was still multilayered). In addition, we observed a somewhat fainter but unambiguous signal in the inner part of the outer granule cell layer and in the nascent internal granule cell layer (Fig 1C and insert in Fig 1C). This became clearly visible from p5 onward, when individual layers of the cerebellar cortex became more prominent and could be easily delineated (Fig 1D–F). At p15, the internal granule cell layer was still positive for Mtss1 (Fig 1G, H). In contrast, at p21 (Fig 1I), and in adult specimens (Fig 1J, K), the Mtss1 signal was restricted to Purkinje cells, and indeed to their perikarya. Even prolonged development of the color reaction did not result in any appreciable signal localized over Purkinje cell dendrites (Fig 1K and data not shown). We did not observe any indication of a differential expression, along the anterior-posterior axis, in vermal sections of all ages analyzed, neither in Purkinje cells, nor in granule cells (Fig 1G, J and data not shown). Moreover, analysis of coronal sections of p3, p8 and p9 animals also showed homogeneous expression along the medio-lateral axis, again in Purkinje cells and in granule cells. I.e., there was no indication that molecularly defined sagittal compartments (e.g., [24,25]) might differ with respect to Mtss1 expression. Control sections hybridized with sense probe did not show any signal (not shown). All of the above results were obtained with a probe derived from the 5'-part of Mtss1 (probe A in Fig 2; extending from exon 1 to 9). In addition, we hybridized cerebella derived from p8 and adult animals with a second in-situ probe derived from the last 3' UTR (probe B; for primers, see additional file 1). Except for slight differences in signal strength which probably relate to probe length, we observed identical results to the ones documented for the 5'-Mtss1 probe (data not shown).

### Differential splicing of Mtss1 during cerebellar development

**Figure 3:**
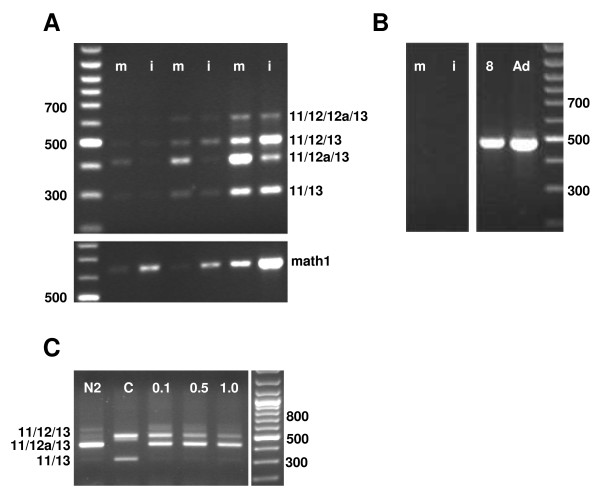
**The relative abundance of Mtss1 comprising either exon 12 or exon 12a changes during neuronal differentiation**. **A, B: **Immature (i) and more mature (m) granule cells were isolated based on their differential expression of Math1-EGFP by flow sorting. Results from 3 independent experiments are shown in panel **A**. In mRNA from immature granule cells, the band indicative of the exon variant 11/12/13 is most prominent. In contrast, in more mature granule cells, the band indictive of the splice variant 11/12a/13 is strongest. Sorting efficacy was verified by screening for expression of endogenous Math1 (lower part of panel A). **B**: contamination of granule cell fractions by Purkinje cells was excluded by screening for the Purkinje cell specific Marker, L7/Pcp2. Lanes 8 and Ad positive controls with mRNA prepared from p8 and adult cerebellum. **C**: In mRNA isolated from P19 cells differentiated by growth in N2 medium (lane N2), the Mtss1-band indicative of the splice variant 11/12a/13 is most prevalent. In mRNA from undifferentiated controls (C), that representing the exon combination 11/12/13 predominates. Upon induction of differentiation with increasing doses of retinoic acid (0.1, 0.5 and 1.0 μM for 4 days), the intensity of the band indicative of usage of exon 12 gradually decreases, whereas that indicative of exon 12a remains unchanged, i.e. there is a relative shift towards the latter. For panel A and C, primers 4 and 5 were used.

Previous studies have revealed the existence of several variants of Mtss1, both in normal murine tissue [12] and in tumor cell lines [18,19]; cf also [26]. We used PCR to further elaborate the molecular structure of the Mtss1 isoform(s) expressed in the cerebellum and purified cell populations thereof. Using primers located in exons 1 and 9 (primers 1 and 3; cf Fig 2 and table in additional file 1), we observed a single PCR product of the size predicted for this segment encompassing all known exons, in cerebella of p8 and adult mice, and also in RNA prepared from immature and maturing granule cells sorted based on their level of Math1-EGFP expression (see below). Specifically, we did not detect a band which might be indicative of an alternative usage of exon 7 (not shown). We verified expression of exon 7 using primers located in exon 7 (i.e., 12 out of 19 nt were from exon 7; primer 2 in the table given in additional file 1) and exon 13 under stringent annealing conditions. This resulted in 4 discrete bands (Fig 2C). The same pattern of bands was seen when primers located in exons 11 and 13 were used (Fig 2D; 3A); these were shifted towards lower molecular weights by what would be expected based on the sequence separating exons 7 and 11, and encompassing all (known) intervening exons. The bands obtained after amplification between exons 11 and 13 were verified by sequencing and found to comprise 292, 412, 487 and 607 nt, respectively. Thus, they correspond to splice variants comprising the exon combinations 11/13, 11/12a/13, 11/12/13 and 11/12/12a/13, respectively. Intriguingly, the relative intensity and pattern of these bands varied for RNAs prepared from different developmental stages (Fig 2D). A developmental pattern identical to the one shown in Fig 2D was consistently obtained in RNA prepared from three sets of mice in three independent experiments. In particular, we consistently observed that band corresponding to the exon combination 11/12/13 was most prominent at p3 and p5. Analysis of peripheral tissues further revealed that the splice variant 11/12a/13 is found exclusively in the CNS (supplemental figure S1A in additional file 5).

We further observed, in the developing p8 and the adult cerebellum, the expression of both the short (163 nt) and the long (209 nt) versions of exon 14 described in the Ensembl databank (supplemental figure S1B, see additional file 5). In addition, we also observed very weak bands at approximately 500 and 550 nt (not visible in supplemental figure S1Bof additional file 5), which probably correspond to a splice variant in which exon 15 is subdivided in a N- and C-terminal part separated by 108nt [12]. Clearly, this variant is expressed at best at very low levels in the murine cerebellum. None of the splice variants downstream of exon 12 were observed to be specific to the cerebellum; nor did we obtain any indication that they might be developmentally regulated. Therefore, we did not follow up on them presently.

### Association of differential splicing of exons 12 and 12a with cellular differentiation

The developmental shift of relative intensities of the bands representing splice variants of the region between exons 11 and 13, in conjunction with the temporal differences of Mtss1 expression in Purkinje and granule cells, prompted us to ask whether these splice variants might be expressed in a cell type-specific pattern. To address this issue, we isolated cerebellar granule cells based on their expression of an EGFP-tagged, Math1-derived transgene [27] by FACS and assessed their expression of Mtss1-splice variants by RT-PCR (Fig 3). We chose this approach rather than attempting to derive splice variant-specific probes for in situ hybridization, because the sensitivity and specificity of such probes would be critically limited by the relatively small sizes of the exons concerned (between 120 and 211 bp), and the problems inherent in quantitative cross specimen comparison of hybridized sections.

In the cerebellar anlage, Math1 is specifically expressed in immature, proliferating granule cells, and it is rapidly down-regulated once these cells stop to proliferate and migrate to their adult position [28]. We capitalized on the fact that this developmental regulation is replicated by a Math1-EGFP transgene, the expression of which quantitatively correlates with granule cell maturation [29], to isolate mRNA from two subsets of granule cells, i.e. immature, strongly Math1-EGFP positive, and maturing granule cells, which express only low levels of EGFP (Fig 3). We assessed the efficacy of this approach by evaluating levels of cognate Math1 mRNA in the cell populations thus obtained (Fig 3A, lower panel). We further verified that the granule cell populations obtained were not contaminated by Purkinje cells by screening for the presence of the Purkinje cell specific marker, L7/Pcp2 [30,31], which could not be detected in any of the granule cell fractions analyzed (Fig 3B and data not shown).

Both immature (strongly Math1-EGFP-positive) and maturing (weakly Math1-EGFP-positive) granule cells expressed the same complement of splice variants seen in mRNA prepared from whole cerebellum. However, the relative intensities of individual bands varied in a systematic and consistent way (Fig 2D). Whereas in the adult cerebellum, where Mtss1 is expressed exclusively in Purkinje cells [12]; and see Fig 1J, K), the band representing exon combination 11/12a/13 is the most prominent one (Fig 2D), the major band seen in immature granule cells is the one indicative of exon combination 11/12/13 (Figs 3 and 3A). In contrast, in mRNA prepared from maturing granule cells, like in that from adult cerebellum, the band indicative of the exon combination 11/12a/13 (Fig 3A) is most prominent. Such a developmentally regulated switch could also be observed in P19 cells induced to differentiate into neural cells (Fig 3C).

**Figure 4:**
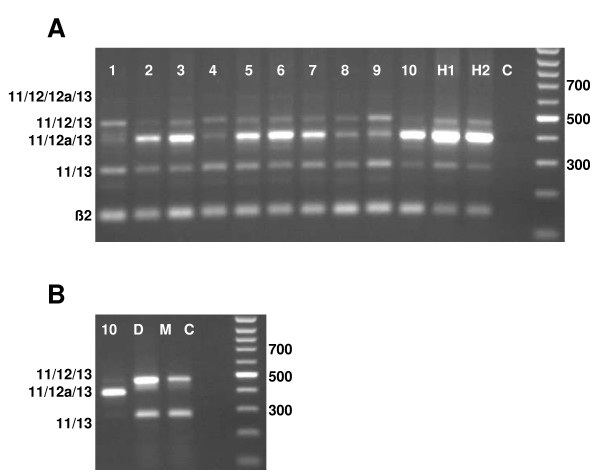
**Expression of Mtss1 exons 11–13 in human medulloblastomas and medulloblastoma derived cell lines**. **A**: Lanes 1–5: classical medulloblatoma samples D1198, D1127, D1049, D1185 and D963, respectively; lanes 6–10: desmoplastic medulloblastoma samples D86, D978, D82, D1401, and D1062; lanes H1, H2: fetal human cerebellar samples R1626 and R1628. Lane C is a negative control. The band indicative of the splice variant containing exons 11/12a/12/13 is hardly visible in this reproduction. **B**: In DAOY (D) and D-283Med medulloblastoma cell lines, bands representing the splice variants 11/12/13 and 11/13 predominate. Note absence of the band indicative of the splice variant 11/12a/13, which should comigrate with the prominent band of sample 10 shown for comparison. Lane C is a negative control.

Cerebellar granule cells, or their precursors, have been identified as a cellular origin of medulloblastomas. Based on their morphology, pattern of gene expression and biology, two major subsets of medulloblastomas have been defined, referred to as classical and desmoplastic types, respectively [32-34]. Analysis of mRNAs obtained from 5 classic and 5 desmoplastic tumor specimens [35] revealed that all of them expressed Mtss1, and specifically the same basic splice variants within the region of exons 11–13 as described above (Fig 4A). Among individual tumor samples, the relative intensities of these bands varied, and again as described for the developing cerebellum, the most conspicuous differences were observed for the bands representing exon combinations 11/12/13 and 11/12a/13. In two cell lines derived from human medulloblastomas (DAOY and D-283Med), exon combinations 11/13 and 11/12/13 were prominent, whereas the combinations 11/12/12a/13 and 11/12a/13 could not be observed (Fig 4B). As expected from the murine data, the most prominent band in mRNA obtained from normal, immature human cerebellar tissue was the one indicative of the exon combination 11/12a/13. The band representing the exon combination 11/12/12a/13 of Mtss1 appeared very weak in all human specimens analyzed.

### Mtss1 exon 12a is conserved in mammals

**Figure 5:**
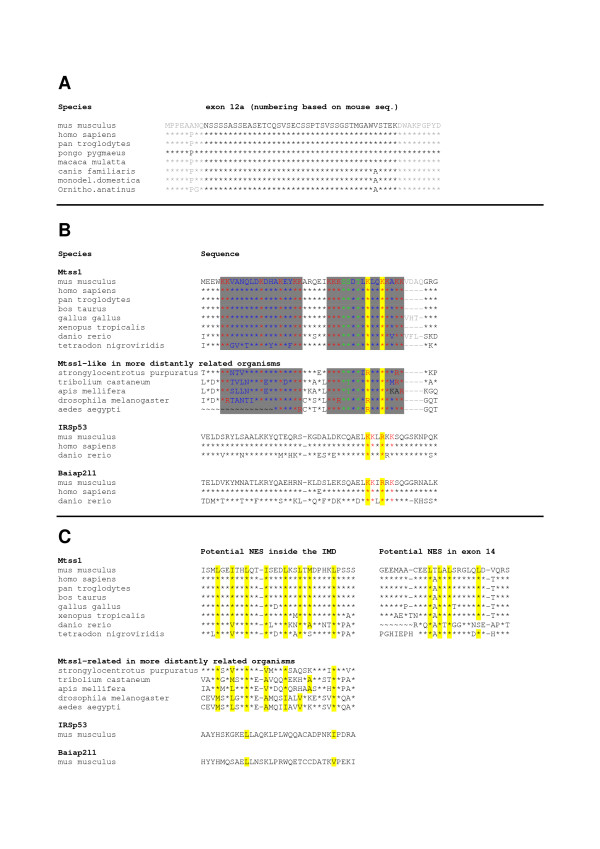
**A: **Sequence comparison of exon 12a of Mtss1 in various mammals. Sequence rendered in grey letters are from adjoining exons. The * identifies conserved amino acids. Exon numbering is based on the murine sequences. **B, C: **Putative nuclear localization (B) and export (C) signals in Mtss1 are evolutionary conserved. **B**: Alignment of part of the IMD domain-sequences of Mtss1 and its structural relatives IRSp53 (also known as Baiap2) and Baiap2l1. The two nuclear localization signals identified are marked by a gray background. Basic amino acids are labeled red, and potential phosphorylation sites are marked green. The basic amino acids binding actin are marked by a yellow background. Note that these are conserved among Mtss1, IRSp53, and Baiap2l1, whereas the nuclear localization signal is found only in Mtss1, and a set of arthropod proteins which share the IMD domain of the Mtss1 type, but diverge from Mtss1 C-terminally. Note also that the nuclear localization signal centered about the basic, actin filament binding motif is immediately adjacent to the four amino acids encoded by exon 7 (shown in gray). **C**: The leucine-rich motif constituting a putative nuclear export signal inside the IMD is highly conserved for Mtss1. It is not found in the structural homologue, IRSp53. Also shown is the phylogenetic conservation of the nuclear export signal (NES) outside the IMD, which, in the mouse, is encoded by exon 14.

A BLAST search of the NCBI nr database using the murine Mtss1 exon 12a as input identified highly conserved, full-length matching sequences in the Mtss1 genes of several mammals (Fig 5A), but not in non-mammalian genomes. Indeed, the sequence and the exon/intron structure of Mtss1 in xenopus tropicalis and danio rerio (cf Ensemble.org) substantially divergence from that of mammal and chick, over the region comprising exons 12 and 12a in these species, such that an alignment of these regions seem not sensibly possible. This contrasts with the highly conserved structure over the IMD, and also over more C-terminal parts of Mtss1 across species.

### Analysis of Mtss1 in silico

To get a first clue as to the potential functional consequences of differential splicing of Mtss1, we compared the various splice variants of Mtss1 observed presently using a bioinformatic approach. Neither of the two known functional domains of Mtss1, i.e. the N-terminal, actin bundling IMD domain [36], nor the actin monomer-binding WH2 domain located close to its C-terminus [11,14] would be (directly) affected in the splice variants reported here. In addition to these well characterized functional domains, Mtss1 contains a serine-rich region, spanning amino acids (aa) 242–363 in the exon 11/12/13 splice variant, i.e. ending in the N-terminal part of exon 12 (which encodes aa 349–414). As exon 12a encodes a rather serine-rich sequence, this serine-rich region is somewhat extended in the exon 11/12a/13 splice variant, where it spans aa 242–379 (ScanProsite). The translation of the short sequence in exon 15 that may variably be interpreted as an exonic or intronic sequence would result in an extension of the proline rich domain in the C-terminal part of Mtss1.

Analysis of the Mtss1 sequence for protein-protein interaction motifs using the algorithms implemented in iSPOT, Scansite, and Minimotif (MnM; [37,38], and by inquiry of the ELM server [39], revealed the occurrence of a number of short peptide motifs predicted to bind to proteins containing SH3, SH2, 14-3-3, WW or FHA domains [40-44] (see tables 2–4 in additional files 2, 3, 4). Intriguingly, two out of a total of four motifs in Mtss1 predicted to bind to the non-receptor tyrosine kinases Fyn and Src are encoded by exon 12. One of these motifs (starting at aa 378, PASRLLPRVT) is predicted to bind to the SH3 domains, the other (centered about Y397, which has actually been observed to be phosphorylated (cf the PhosphoSite database [45]; and [46]) to SH2 domains of Fyn and Src, and both received reasonably high prediction scores for these interactions (additional files 2 and 3; compare with the score of 0.6903 for cortactin, which has been experimentally verified to bind Mtss1 [14]). There is only one additional SH3-binding motif outside of exon 12 in Mtss1 predicted to bind Fyn and Src (starting at aa 644, in the N-terminal part of exon 15), and one SH2-binding motif predicted to bind Fyn (centered about Y260).

Exon 12a is predicted to form one (out of a total of 16 in the complete sequence of Mtss1) class IV WW binding motif, and one (out of a total of 9) FHA-domain binding motif (additional file 4). The functionality of these motifs, which need to be phosphorylated for binding, remains unresolved as we have no information about their phosphorylation.

We complemented this prediction of potential protein binding motifs in Mtss1 by an extensive search of the literature and the BGEM [47] and Allan Brain Atlas [48] databases for information about the cerebellar expression, potential function, and pathophysiological changes of the highest scoring potential Mtss1-interaction partners thus identified. The results are summarized in supplemental tables 2 and 3 (columns 'Allen', 'BGEM p7' and BGEM Ad'; cf additional files 2 and 3).

**Figure 6:**
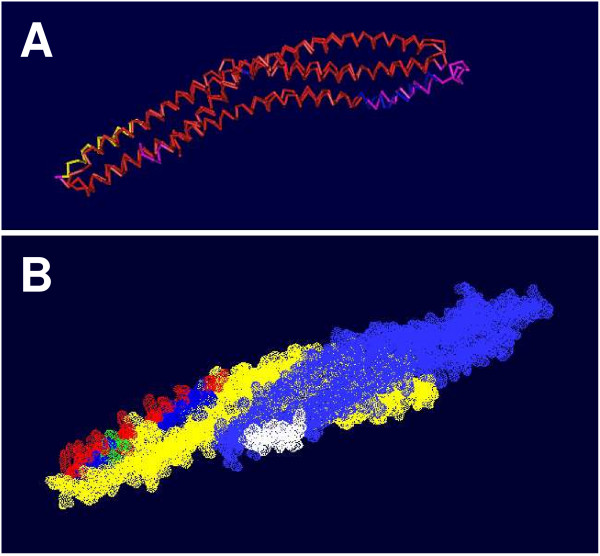
**Structure of the Mtss1 IMD-domain**. **A**: Structural superposition of the IMD domain monomer from Mtss1 (PDB entry 2D1L) with that of IRSp53 (PDB entry 1Y2O; cf [54]. 3D-structures were compared using the Vector Alignment Search Tool (VAST), and visualized with Cn3D 4.1. Alignment is color-coded (red, high), except for the region containing the nuclear localization signal, which is marked yellow to facilitate orientation. **B**: The putative nuclear localization signals (NLS; red, basic amino acids) in the IMD are marked on the chain of the IMD domain pointing to the left, whereas the putative export signal is marked white on the chain pointing to the right. Potential target sites for phosphokinases within/close to the NLS are marked green, whereas the remaining amino acids in the NLS are marked blue (see Fig 5B for sequence details). Panel B was generated using the SwissPDB viewer.

Lastly, comparative analysis of the Mtss1 sequence also revealed the existence of two potential nuclear export signals (a leucine rich sequence in exon 14; **L**T**L**A**L**SRG**L**Q**L**D**V**QRSSRDS**L; **cf also [20] and a hydrophobic segment inside the IMD, aa 223–235; **L**QT**I**SED**L**KS**L**T**M **; (analyzed using NetNES; [49]; Fig 6B; see also scheme in Fig 2B). In addition, two bona fide, bipartite nuclear localization signals (NLS; [50,51]) could be recognized in Mtss1 (one starting at aa 116, KKVANQLDKDHAKEY**KK**, and one at aa 138, **KK**KSSDTL**K**LQ**KK**AKKV, respectively; identified using the PSORT II server [52]). Both of these nuclear localization signals are located in the IMD domain (Fig 2B; Fig 6A, B). Intriguingly, both NLS comprise amino acid residues previously found critical for actin filament binding by Mtss1 (the so called basic patch; boldfaced in the above sequences), some of which are also conserved in the IMD of IRSp53 (underlined and boldfaced in the above sequences) [15,16,53,54]. In this context, it seems worth mentioning that S141, S142, as well as T144 are predicted as potential phosphorylation sites for PKC, PKA, and calmodulin dependent protein kinase 2 (Scansite, ELM, PPsearch). Both predicted basic nuclear localization signals, and the presumed nuclear export signal in the IMD, are highly conserved in Mtts1 from various species, and also in a set of proteins from species which express proteins that share a considerable degree of homology with Mtss1 in IMD domain, but not beyond (Fig 5B, C). Similarly, the nuclear export signal localized to exon 14 of the murine sequence is also highly conserved in Mtss1 across species (Fig 5C). No comparable sequences are found in the IMD-domain containing proteins IRSp53 and Baiap2l1 (Fig 5B, C).

## Discussion

### Expression and splicing of Mtss1 during neuronal differentiation

The protracted time course of cerebellar histogenesis and the spatial segregation of progressively differentiated neurons within the cerebellar cortex allowed us to relate the developmental expression of Mtss1, in cerebellar granule cells, to the postmitotic migration and neuritogenesis of these cells [55-57]. Once they settle in the internal granule cell layer and get synaptically integrated, they cease to express Mtss1. Progressive granule cell differentiation is further accompanied by a switch in Mtss1 splicing, such that usage of exon 12 is down-regulated, whereas expression of exon 12a increases strongy. An identical switch can be triggered in P19 cells upon their induction to differentiate into neurons. Together with the observation that exon 12a is not expressed in peripheral tissues, this indicates that the switch from exon 12 to 12a usage occurs specifically in neurons and is linked to their terminal differentiation.

As a cautionary note, we would like to stress that differences in the intensities of the PCR bands representing splice variants comprising exons 12 and 12a respectively cannot be directly equated with absolute mRNA levels, let alone levels of the corresponding protein isoforms. Rather, comparative assesment of band intensities as used here may be conceptually and methodologically likened to aproaches using exogenous competitors to (semi-)quantify mRNAs (e.g., [58,59]). Differences in PCR efficacy, and in vivo, potential differences in translational regulation related to the differentially spliced exons need to be considered. These issues will require the development of reliable tools to distinguish, at the protein level, Mtss1 isoforms arising consequent to differential splicing. Still, our data document that exon 12a is expressed, at detectable levels, only in nervous tissue, and that its expression increases with neuronal differentiation; in contrast in cerebellar granule cells, as in the forebrain (our unpublished observations), expression of Mtss1 comprising exon 12 ceases concomitant with neuronal differentiation. We directly relate relative band-intensities of defined splice variants to each other, an approach that is equivalent to

The continued, life-long expression of Mtss1 in Purkinje neurons, which contrasts to its differentiation-associated down-regulation in cerebellar granule cells, and indeed throughout the CNS (unpublished observations; and compare expression data in the BGEM and Allan Brain Atlas databases), suggests a role, in Purkinje cells, beyond neural migration and neuritogenesis. It is tempting to relate continued Mtss1 expression in Purkinje cells to their functional plasticity, realized at the level of their dendritic spines, which remain mobile throughout life [60,61]. This mobility is known to be actin-dependent [60,62,63]; see also [64].

#### Possible consequences of the differential usage of exons 12 and 12a

The precisely timed and tissue-specific change in the usage of exons 12a and12 during neuronal differentiation begs the question as to its potential significance. The part of Mtss1 encoded by the region encompassing these exons is evolutionary quite variable, in contrast to the rather strong conservation of the IMD domain, and also the C-terminal part of Mtss1. Indeed, from an evolutionary perspective, exon 12a appears to be rather novel, as no homologous sequences are found in the Mtss1 genes of xenopus and zebrafish.

Of the several motifs predicted to mediate protein-protein interactions encoded by exons 12 and 12a, those predicted to mediate interaction with fyn and src, which are only encoded by exon 12, but not by exon 12a, appear particularly intriguing. Indeed, the combined ablation of Fyn and Src in the CNS has been reported to cause a reeler-like phenotype [65], i.e. a migratory defect that also affects cerebellar histogenesis. Thus, Mtss1 might constitute part of the link that relates activity/subcellular localization of these kinases to morphogenesis. The developmental switch in the use of exons 12 and 12a may be expected to substantially affect interaction of Mtss1 with Src and Fyn, or at least lead to the generation of two isoforms with differential. Finally, we note that the exchange of exon 12 for 12a may also affect interaction with Gli, as exon 12 (which encodes aa 350–414) codes part of a broad region (aa 160–399) to which the interaction site for Gli1 has been localized [20].

### A structural rationale to coordinate actin bundling and nuclear translocation

Mtss1 has been observed to localize both to the cytoplasm and nuclei in fibroblasts [13], and indeed the latter is a key prerequisite for its interaction with Gli proteins in transcriptional regulation as observed by Callahan et al [20]. The identification, here, of two evolutionary highly conserved nuclear localization motifs and an equally conserved nuclear export motif suggests a structural rationale for a twin role of Mtss1 as a cytoskeleton associated protein and a transcriptional modulator/regulator. As detailed above, the actin-binding "basic patch" [53] of the Mtss1-IMD domain is integrated in a bona fide bipartite nuclear localization signal, which suggests dual use of this element. Several potential phosphorylation sites intercalated within this nuclear localization motif further suggests that use of this element might be dynamically regulated. Finally, phosphorylation of T144, which is part of a predicted PKCα/β/γ phosphorylation site (Scansite score 0.3985; percentile 0.183), would transform the actin basic patch/nuclear localization motif in a perfect FHA domain-binding motif (ELM). This domain is found in many transport-associated and nuclear proteins (cf above). That this view is conceptually on target is corroborated by the recent observation that deletion of a larger part of the IMD domain (aa 108–153), which included the actin binding basic patch/nuclear localization motif/FHA-binding motif identified here, indeed affects Mtss1 subcellular distribution [46].

Thus, if we allow for a somewhat speculative perspective for the moment, we might envision the basic patch/nuclear localization motif in the IMD domain of Mtss1 could be critical for a dynamic, activity-dependent relay, triggered by (synaptic) input at the spine, and formed by the spine actin cytoskeleton, kinesins, the nuclear import machinery, and lastly nuclear partners of Mtss1. This perspective is also based on the observations that Rac, a known binding partner of Mtss1 [66,67] (cf also [68]) is critical for Purkinje cell spine morphogenesis [69]; so is IRSp53 [70], which also functions as a downstream signal tranducer of insulin-like growth factor 1, an exemplary regulator of Purkinje cell dendritic plasticity [71]. The IMD domain shard between IRSp53 and Mtss1 begs the question whether they might interact, or compete for binding partners. Finally, our in silico approach suggests Crk and Esp8 as potential interacting proteins for Mtss1, both of which have been linked to cerebellar development and function [72-74] (cf also [75]).

## Conclusion

The present findings document the developmentally regulated splicing and expression of Mtss1 in cerebellar granule and Purkinje neurons. They suggest that these isoforms of Mtss1, which differ in (a) domain(s) predicted to mediate interaction with a set of proteins known to affect the physiology of Purkinje and granule cells, may be related to specific functional characteristics and properties of these cells, in particular developmental migration and adult synaptic plasticity. Lastly, bioinformatic analysis of Mtss1 suggests that the basic patch of the IMD domain, which mediates actin binding, overlaps with a nuclear localization signal and suggests a key structural motif that may be regulated in an activity-dependent manner to define subcellular distribution, and hence function, of Mtss1. Our findings define the cerebellum, and Purkinje cells in particular, as a paradigm to further test the function of Mtss1, to unravel how its functional properties are structurally encoded, and how they may relate to tumor cell biology.

## Methods

### Animals and tissue preparation

All animal handling was done in strict adherence to local governmental (European Communities Council Directive 86/609/EEC) and institutional animal care regulations. Cerebellar tissue for in situ hybridization or mRNA prepared from whole cerebella was obtained from C57Bl/6J mice of defined ages. Granule cell precursors were isolated from transgenic mice expressing EGFP under control of a Math1-derived promoter [29]. For the preparation of tissue, mice were deeply anesthesized by intraperitoneal injection of avertin (2,2,2-tribromoethanol; 0.03–0.06 ml/g body weight of a 2.5% solution) and killed by cervical dislocation. To prepare cerebella for in situ hybridization, brains were dissected, rinsed with phosphate-buffered saline (PBS; 150 mM NaCl, 10 mM NaH_2_PO_4_; pH 7.2), frozen on dry ice, and subsequently stored at -80°C. Fifteen-micrometer cryosections were obtained at -20°C, taken up on slides at room temperature (RT) and stored at -80°C until further use. They were fixed for 5 min in 4% paraformaldehyde immediately prior to the hybridization procedure, which was performed as described [76]. Hybridized probes were visualized using the alkaline phosphatase system with BM-purple as chromogen (Roche, Mannheim, FRG). Sections were counterstained by incubation in Hoechst 33342 (1 mg/ml in PBS) for 5 min, followed by two short washes in PBS and then coverslipped with mowiol (Merck, Darmstadt, FRG. Micrographs were obtained using an Axioskope 2 microscope (Zeiss, Oberkochen, FRG) and a digital camera (DT5; Olympus, Hamburg, FRG). Images were arranged using Adobe Photoshop software.

DAOY [77] and D-283Med cells [78]were grown under standard conditions.

### Probe preparation

A 718 nucleotide (nt) long Mtss1-specific probe was generated by PCR using primers located in exons 1 and 9 (see Fig 2; primer sequences are given in additional file 1). Sense and antisense labeled cRNAs were obtained from T3/T7 flanked PCR-products by in vitro transcription as described [79]. A second, 606 nt long probe was generated using primers located in the 3'UTR.

### RNA isolation and PCR

To isolate RNA from whole cerebella, postnatal (from postnatal day 0 (P0), P3, P5, P8, P15 and adult) cerebella were dissected under sterile and RNase-free conditions in PBS. They were carefully freed of meninges, and then frozen in liquid nitrogen and stored at -80°C until use.

To isolate precursors of granule cells or cerebellar cortical inhibitory interneurons, we prepared, by combined mechanical and tryptic digestion [80], a single cell suspension from cerebella of transgenic mice expressing the enhanced green fluorescent protein (EGFP) under control of a Math1-derived promoter [29]. Briefly, cerebella were dissected in modified Hank's balanced salt solution (4.17 mM NaHCO_3 _and 0.7 mM Na_2_HPO_4_, pH 7.2). They were carefully freed of their meninges, and cut into small pieces which were then incubated with trypsin (0.1% in phosphate-buffered saline (PBS) containing 1.06 mM EDTA) for 15 min at 37°C. Trypsinization was stopped by adding PBS containing 8 mg/ml trypsin inhibitor and 8 mg/ml bovine serum albumin, and the resulting cell slurry was sequentially passed through nylon meshes with a pore diameter of 250 and 30 μm, respectively. Cells were pelleted by centrifugation (300 g; 10 min) and then resuspended at a density of 5*10^6 ^cells/ml in phenol red-free Neurobasal medium (Invitrogen, Karlsruhe, FRG) supplemented with B27 (2%, v/v; Invitrogen) and 2 mM glutamax (Invitrogen). The resulting cell solution was passed twice trough a 20 G needle. Subsequently, propidium iodide was added at a final concentration of 0.5 μg/ml. Immediately before sorting, cells were filtered once more by passage through a 40 μm pore size mesh (TechFab, Geldern, FRG). They were then sorted in Hank's balanced salt solution (Invitrogen) on a FACSDiva cell sorter (BD Biosciences, Heidelberg, FRG) using the 488 argon laser line for excitation. EGFP fluorescence was recorded using a 520/20 bandpass filter, and propidium iodide uptake was monitored using a 630/22 bandpass filter. Viable (i.e., propidium-iodide negative) cells were sorted based on their level of EGFP expression and collected in neurobasal medium (Invitrogen) supplemented with 10% bovine serum albumin. They were concentrated by centrifugation (300 g; 10 min), shock frozen in liquid nitrogen and stored at -80°C until further use.

Total RNA was prepared from frozen cells/cerebella trizol (Qiagen, Hilden, FRG; for whole cerebella). The resulting RNA samples were digested with RNase-free DNase I (Qiagen). Total RNA was quantified photospectrometrically at 260 nm, and 5 μg of total RNA was reverse transcribed using 200 U of RNase H reverse transcriptase (Superscript II; Life Technologies, Karlsruhe, FRG) in a volume of 20 μl that also contained oligo dT primers (Life Technologies) at 0.5 μg/μl. One hundred nanograms of the resulting cDNA was then amplified in a final volume of 25 μl with 1× PCR buffer which contained 1.5 mM MgCl_2_, 200 μM of each dNTP (Fermentas, St. Leon-Rot, FRG), 10 pmol of both forward and reverse primers (Invitrogen), and 1.54 U *Taq *DNA polymerase (Fermentas). Amplification was carried out following a denaturing step for 3 min at 94°C with 30–32 PCR cycles as follows: 94°C for 30 s, 57°C for 30 s, 72°C for 1 min, and a cooling step at 4°C. The primers used and the expected size of the reaction products are given in Table 1. In all experiments cDNA was normalized to GAPDH or β2-microglobulin mRNA levels. PCR products were analyzed on 1% agarose gels stained with ethidium bromide (0.2 μg/ml), viewed under UV light and recorded with a BioRad (Munich, FRG) gel documentation system.

Selected PCR products were cloned in a pBluescript II SK(+) derivative modified for T/A cloning [81], and the cloned amplification products were verified by sequencing (Seqlab, Göttingen, FRG).

### Cells culture

P19 cells were grown in Dulbecco's modified Eagle's medium containing 10% fetal calf serum. To induce differentiation, cells (10^7^/ml) were allowed to aggregate in bacterial culture dishes in the presence of retinoic acid at the doses indicated under results. After 4 days, cells were collected, trypsinized, and seeded onto poly-L-lysine (20 μg/ml) coated tissue culture dishes. They were grown in N2 medium for 4 to six days. Differentiation status was checked by phase contrast microscopy. For RNA preparation, adherent cell were directly lysed in trizol.

### Human tumor cDNA samples

cDNA samples of human medulloblastomas and normal human fetal cerebellum were obtained from patients enrolled in the multicenter treatment study for pediatric malignant brain tumors (HIT) of the German Society of Pediatric Hematology & Oncology (GPOH). Details of these samples have been described previously [35].

### Bioinformatics procedures

For an initial bioinformatic analysis of Mtss1, we inquired the following on-line data sources, and/or applied methods implemented in on-line services available at these sites: iSPOT, [37]; Scansite [82]; the ELM server, [39]; Minimotif (MnM; cf [38]; the SMART server [84]; the PPsearch protein motif search (implemented at [85]); NetPhos2.0 and NetNes [49,83]; the PhosphoSite 1.5 database [45]. Pfam, MultAlin and ScanProsite were accessed through the ExPASy proteomics server of the Swiss Institute of Bioinformatics (SIB; cf [86]). Protein structural data were obtained from the PDB database [87] and the VAST database [88], and viewed using Cn3D 4.1 [89] and the Swiss PDBViewer 3.7 [90], which were also used for the preparation of molecular structure images. Further, we screened the Brain Gene Expression Map database (BGEM; cf [47] and the Allen Brain Atlas (Allen Institute for Brain Science; cf [48]) for expression data of genes of interest.

## Competing interests

The author(s) declares that there are no competing interests.

## Authors' contributions

AG and SM carried out in situ hybridizations, PCR-experiments, and cell culture studies. LS and TAN contributed to the in-situ hybridization experiments and histological analyses. WH and TP collected human tissue samples and analyzed them. MH, SLB, JO, TP and KS contributed to the bioinformatical analyses; SLB, JO and TP contributed to the design of the study. KS conceived of the study, designed and coordinated it and drafted the manuscript. All authors read and approved the final manuscript.

## Supplementary Material

Additional file 1 List of primers used. Primers used in PCRs documented in figures 2, 3, 4 and in supplemental figure S1 (additional file 5). Click here for file

Additional file 5 Supplemental figure S1: Expression of Mtss1 splice variants in the early postnatal and adult cerebellum and in peripheral murine tissues. The data document Mtss1 splice variants found in the developing and adult cerebellum and in various non-neural tissues. Click here for file

Additional file 2 SH3-domain binding motifs found in Mtss1. List of potential SH3-domain binding motifs identified in Mtss1. Click here for file

Additional file 3 SH2-domain binding motifs found in Mtss1. List of potential SH2-domain binding motifs identified in Mtss1. Click here for file

Additional file 4 Binding motifs for 14-3-3-, FHA- and WW class IV-domain proteins in Mtss1. Potential binding motifs identified in Mtss1 for proteins with 14-3-3-, FHA- or WW class IV domains. Click here for file
