# Supplementary material for: Developmental expression and differentiation-related neuron-specific splicing of metastasis suppressor 1 (Mtss1) in normal and transformed cerebellar cells
Source: BMC Dev Biol. 2007 Oct 9;7:111. doi: 10.1186/1471-213X-7-111 (PMC2194783; doi:10.1186/1471-213X-7-111)
Supplement: Additional file 1 — List of primers used. Primers used in PCRs documented in figures 2, 3, 4 and in supplemental figure S1 (additional file 5). [file 1471-213X-7-111-S1.doc]

**Supplemental table 1**

PCR primers used

| **number**  **(Fig 3)** | **primer code** | **orient.** | **sequence** | **length** | **Mtss1**  **Exon** |
| --- | --- | --- | --- | --- | --- |
| 1 | m-Mtss1-24131 F | f | GGAGGCTGTGATCGAGAAG | 19 | 1 |
| 2 | m-Mtss1Ex7Forw | f | AAAAAGTGGACGCTCAGGG | 19 | 7 |
| 3 | m-Mtss1-24131 R | r | CATGGACCCTCACAAGCTG | 19 | 9 |
| 4 | m-Mtss1Ex11Forw. | f | AACAGCGTCAACAGCAGTG | 19 | 11 |
| 5 | m-Mtss1Ex13Rev | r | CCCCATTAGAGTCTGGCTC | 19 | 13 |
| 6 | m-Mtss1Spl. Forw. | f | AGCTGGACGTGCAGAGG | 17 | 14 |
| 7 | m-Mtss1-24131+42632 F | f | AAGACGACCACCAACGATC | 19 | 15 |
| 8 | m-Mtss1 Spl. Rev. | r | TCTCTGGAATCGCCTGTC | 18 | 15 |
| 9 | m-Mtss1-24131+42632 R | r | GAGAAGGAAACCGGACATG | 19 | 3'UTR |
| probe A | m-Mtss1-F-T3-24131 | f | aagcttaattaaccctcactaaagggGGAGGCTGTGATCGAGAAG | 45 | 1 |
| probe A | m-Mtss1-R-T7-24131 | r | ggatcctaatacgactcactatagggCAGCTTGTGAGGGTCCATG | 45 | 9 |
| probe B | m-Mtss1-42632 F | f | TCCTCTTGACCGCACACAC | 19 | 3'UTR |
| probe B | m-Mtss1-42632 R | r | TGTTCAAAGGGCAAGGCCC | 19 | 3'UTR |
|  |  |  |  |  |  |
| - | human-Mtss1Ex11For | f | AACAGTGTCAACAGCAGTG | 19 | 11 |
| - | human-Mtss1Ex13Rev | r | CGGTTCTCGCTTCTCTTTG | 19 | 13 |
| - | h-beta2-microglobulin-F | f | TGTCTTTCAGCAAGGACTGG | 20 | - |
| - | h-beta2-microglobulin-R | r | GATGCTGCTTACATGTCTCG | 20 | - |
| - | m-Math1-F | f | CCAGCAAACAGGTGAATGGGGTAC | 24 | - |
| - | m-Math1-R | r | CAGGGAGCTGTTGCCTTCCTAACTG | 25 | - |
| - | m-L7/Pcp2-F | f | GATTCTTAGTACTGTCCCCCAAG | 23 | - |
| - | m-L7/Pcp2-R | r | TTATTGTTTTCAGGGGCCAGTG | 22 | - |

Names of primers for murine sequences start with an "m"; those for human sequences with an "h".
