# Supplementary material for: Developmental expression and differentiation-related neuron-specific splicing of metastasis suppressor 1 (Mtss1) in normal and transformed cerebellar cells
Source: BMC Dev Biol. 2007 Oct 9;7:111. doi: 10.1186/1471-213X-7-111 (PMC2194783; doi:10.1186/1471-213X-7-111)
Supplement: Additional file 5 — Supplemental figure S1: Expression of Mtss1 splice variants in the early postnatal and adult cerebellum and in peripheral murine tissues. The data document Mtss1 splice variants found in the developing and adult cerebellum and in various non-neural tissues. [file 1471-213X-7-111-S5.ppt]

## Slide 1
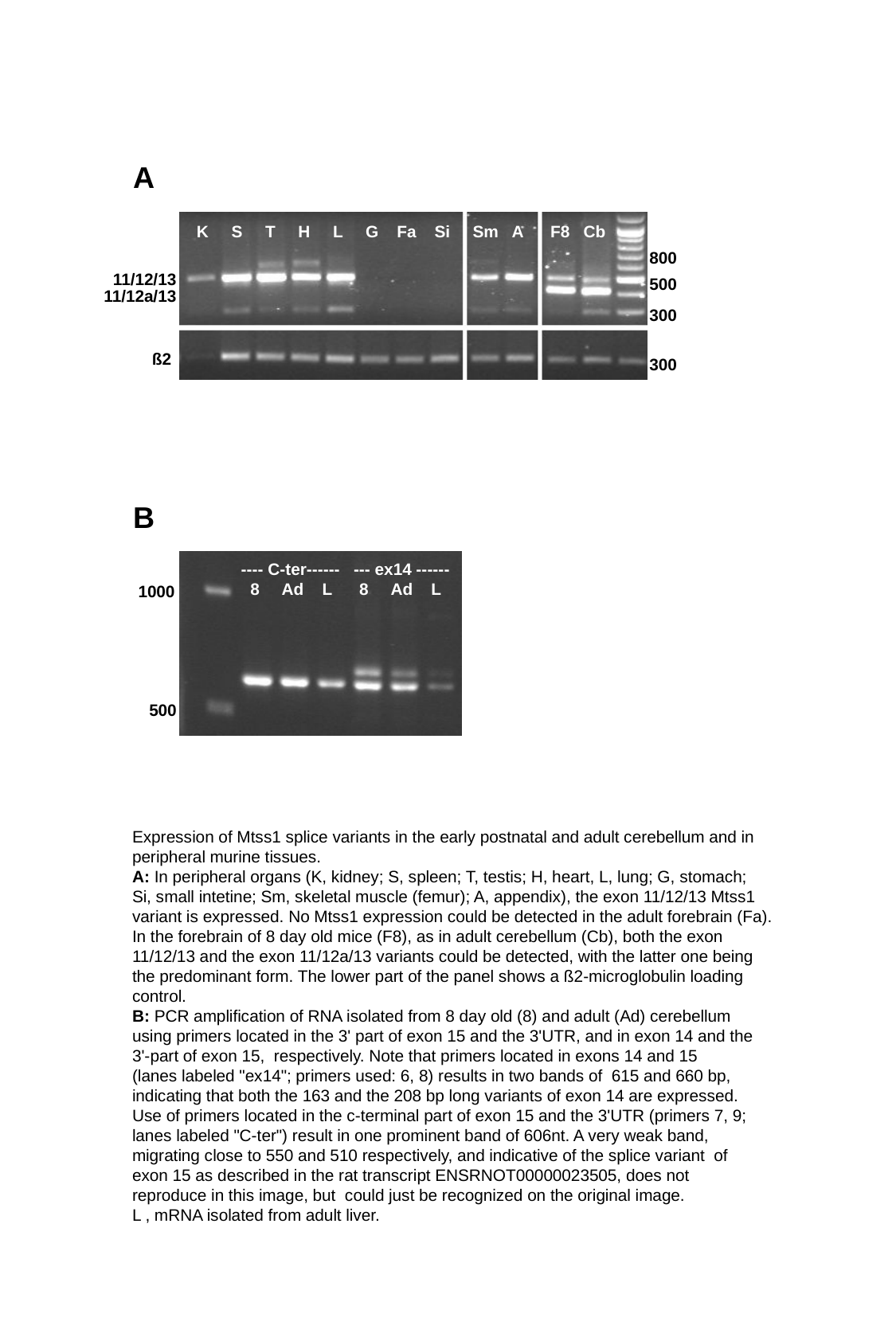

A
K S T H L G Fa Si Sm A F8 Cb
800
11/12/13
500
11/12a/13
300
ß2
300
B
---- C-ter------ --- ex14 ------
 8 Ad L 8 Ad L
1000
500
Expression of Mtss1 splice variants in the early postnatal and adult cerebellum and in
peripheral murine tissues.
A: In peripheral organs (K, kidney; S, spleen; T, testis; H, heart, L, lung; G, stomach;
Si, small intetine; Sm, skeletal muscle (femur); A, appendix), the exon 11/12/13 Mtss1
variant is expressed. No Mtss1 expression could be detected in the adult forebrain (Fa).
In the forebrain of 8 day old mice (F8), as in adult cerebellum (Cb), both the exon
11/12/13 and the exon 11/12a/13 variants could be detected, with the latter one being
the predominant form. The lower part of the panel shows a ß2-microglobulin loading
control.
B: PCR amplification of RNA isolated from 8 day old (8) and adult (Ad) cerebellum
using primers located in the 3' part of exon 15 and the 3'UTR, and in exon 14 and the
3'-part of exon 15, respectively. Note that primers located in exons 14 and 15
(lanes labeled "ex14"; primers used: 6, 8) results in two bands of 615 and 660 bp,
indicating that both the 163 and the 208 bp long variants of exon 14 are expressed.
Use of primers located in the c-terminal part of exon 15 and the 3'UTR (primers 7, 9;
lanes labeled "C-ter") result in one prominent band of 606nt. A very weak band,
migrating close to 550 and 510 respectively, and indicative of the splice variant of
exon 15 as described in the rat transcript ENSRNOT00000023505, does not
reproduce in this image, but could just be recognized on the original image.
L , mRNA isolated from adult liver.
