# Supplementary material for: Developmental expression and differentiation-related neuron-specific splicing of metastasis suppressor 1 (Mtss1) in normal and transformed cerebellar cells
Source: BMC Dev Biol. 2007 Oct 9;7:111. doi: 10.1186/1471-213X-7-111 (PMC2194783; doi:10.1186/1471-213X-7-111)
Supplement: Additional file 2 — SH3-domain binding motifs found in Mtss1. List of potential SH3-domain binding motifs identified in Mtss1. [file 1471-213X-7-111-S2.doc]

# Supplemental table 2

SH3-domain binding motifs found in Mtss1

| **Motif start** | **Sequence** | **score** | **class** | **domain** | **Allen** | **BGEM p7** | **BGEM Ad** | **Refs** | **selected functions** |
| --- | --- | --- | --- | --- | --- | --- | --- | --- | --- |
| 691  639  642  688  632 | NPPLPGPKPS  TPTVPDLPGV  VPDLPGVLPS  APVNPPLPGP  TPVIPVKTPT | 0.7918  0.6724  0.6700  0.6619  0.6374 | 2  2  2  2  2 | ABL_MOUSE | Pj+; gc(+) | egl, igl, Pj? | gc, Pc? | [1-4] | neuronal migration, actin rearrangement causative for dendritogenesis; postsynaptic role in synaptogenesis; dendrite maintenance |
| 644  644  644 | DLPGVLPSPP  DLPGVLPSPP  DLPGVLPSPP | 0.7185  0.7113  0.7311 | 1  1  1 | RUK-ADAPTOR-PROTEIN RAT 101-156 | Pj+ | egl(+) | neg | [5] |  |
| 691  691  642 | NPPLPGPKPS  NPPLPGPKPS  VPDLPGVLPS | 0.6691  0.7254  0.6370 | 2  2  2 | VAV_MOUSE 617-660  VAV_MOUSE 782-842  VAV_MOUSE 782-842 | neg | no data | no data | [6] |  |
| 644  691 | DLPGVLPSPP  NPPLPGPKPS | 0.6796  0.7219 | 1  2 | VAV2_MOUSE 576-642  VAV2_MOUSE 806-867 | neg | no data | no data | [7] | neurite outgrowth in PC12 cells |
| 691 | NPPLPGPKPS | 0.7108 | 2 | ERG13MOUSE (= eps8l2) | Pcl+ | no data | no data | [8,9] |  |
| 644  644 | DLPGVLPSPP  DLPGVLPSPP | 0.6957  0.6616 | 1  1 | GRB2_MOUSE 1-55  GRB2_MOUSE 159-212 | Pj+ | no data | no data | [10] | links EGFR to cytokeleton |
| 644  691  632 | DLPGVLPSPP  NPPLPGPKPS  TPVIPVKTPT | 0.6953  0.6857  0.6772 | 1  2  2 | CRK_MOUSE 132-192 | Pj+++  so  so | egl, igl, Pj? | no data | [11,12] | gc axon elongation; interaction with fyn; link to lissencephaly |
| 691  632 | NPPLPGPKPS  TPVIPVKTPT | 0.6946  0.6543 | 2  2 | ITK_HUMAN | pj very weak | no data | no data | [13] | no documented cns action |
| 644 | DLPGVLPSPP | 0.6903 | 1 | SRC8_MOUSE (cortactin) | Pcl+ (Pj+) | no data | no data | [14,15] | direct interaction with Mtss1 documented; spine morphogenesis |
| 691  644 | NPPLPGPKPS  DLPGVLPSPP | 0.6439  0.6790 | 2  1 | NCK2-SH31 (Nck-beta)  NCK2-SH32 | no exp in cbl | egl++,igl(+), Pj? | no data | [16,17] | Dab1 interacting, reelin pathway; links extracellular signal to actin cytoskeletal remodeling |
| 644  311 | DLPGVLPSPP  AQQAPVRLSS | 0.6774  0.6606 | 1  2 | AMPH_HUMAN | pcl+ | no data | no data | [18] | interference with postsynaptic amphiphysin in Pjs blocks LTD expression |
| 378  644 | PASRLLPRVT  DLPGVLPSPP | 0.6481  0.6735 | 1  1 | FYN_MOUSE | Pcl+ | egl+, igl+ | not informative | [19,20] | reelin pathway; ethanol damage |
| 644 | DLPGVLPSPP | 0.6733 | 1 | PIG1_HUMAN (phospholipase Cgamma) | Pj+; gcl(+) | no data | no data | [21] | mediates BDNF potentiation of GABA signaling |
| 644  691 | DLPGVLPSPP  NPPLPGPKPS | 0.6702  0.6546 | 1  2 | NEBULIN | Pcl(+) | no data | no data |  | muscle cell specific; affected in mdx |
| 644  644  691  644  691 | DLPGVLPSPP  DLPGVLPSPP  NPPLPGPKPS  DLPGVLPSPP  NPPLPGPKPS | 0.6676  0.6506  0.6449  0.6396  0.6386 | 1  1  2  1  2 | NCK1-SH32 (Nck-alpha)  NCK1-SH33  NCK1-SH31  NCK1-SH31  NCK1-SH33 | gca. few cells | no data | no data | [16] |  |
| 691  691 | NPPLPGPKPS  NPPLPGPKPS | 0.6437  0.6655 | 2  2 | EPS8_MOUSE | Pcl(+) | igl+; Pj? | gcl+; pj? | [9,22] |  |
| 378  644 | PASRLLPRVT  DLPGVLPSPP | 0.6621  0.6555 | 1  1 | SRC_HUMAN | Pj++ | egl+; igl+; Pj? | Pcl, igl(+) | [19] |  |
| 716 | DQERDPPSAT | 0.6591 | 1 | JIP1b (Mapk8r1) | Pcl+, Gcl+ | no data | no data | [23-25] | JNK pathway, axon guidance, amyloid metabolism |
| 644 | DLPGVLPSPP | 0.6548 | 1 | HCK_HUMAN | Pcl(+) | no data | no data |  |  |
| 644  691 | DLPGVLPSPP  NPPLPGPKPS | 0.6546  0.6527 | 1  2 | LYN_HUMAN | Pj+ | very weak egl, igl | gcl, Pj? | [26,27] | transiently in Pjs assoc with dendritogenesis; gcs weak egl, stonger igl, incr. with maturation; mediates AMPA signaling |
| 644 | DLPGVLPSPP | 0.6439 | 1 | P85A_HUMAN (Pik3r1, phosphatidylinositol 3-kinase, regulatory subunit, polypeptide 1 (p85 alpha) | Pcl+; gcl+ | pcl+; igl+ | no data | [28,29] | insulin /IGF signaling, neuronal migration |
| 258  688 | WSYQTPPSSP  APVNPPLPGP | 0.6390  0.6420 | 1  2 | SPCN_HUMAN (fodrin; spectrin alpha 2 chain, spna2 (mouse) | Pc+++ | egl(+); igl+; Pj+ | igl+; Pcl++ | [30] |  |
| 644 | DLPGVLPSPP | 0.6345 | 1 | YES_HUMAN | Pj(+) | egl++; igl+; pj? | Pj+ Gcl(+) | [31] |  |

Sequence numbering gives the start of the motif sequence and refer to the sequence of Mtss1 containing exon 12, the N-terminally extended version of exon 14, and the complete (i.e., unspliced) variant of exon 15 (cf Fig 2). Prediction scores are those of iSPOT (best is 1, worst is 0). Column 4 gives the type (class) of SH3 domain preferred.

The columns labeled 'Allen', 'BGEM p7' and 'BGEM Ad' summarize expression data obtained from the Allan Brain Atlas and the BGEM database for 7 day old or adult mice, respectively. Abbreviations used: Pj, Purkinje cell; Pcl, Purkinje cell layer (without unambiguous identification of cell type; gc, granule cell; egl, igl, external and internal granule cell layer, respectively; gcl, granule cell layer. Expression strength is scored from weak (+) to very robust, ++.

In the last two columns, we give key references for neural or cerebellar functions of domain-containing proteins which were identified as potential interaction partners of Mtss1, along with keywords describing these functions or associated pathologies.

## References cited in supplemental tables 2 and 3

1. Finn AJ, Feng G, Pendergast AM: **Postsynaptic requirement for Abl kinases in assembly of the neuromuscular junction.** *Nat Neurosci* 2003, **6:**717-723.

2. Moresco EM, Donaldson S, Williamson A, Koleske AJ: **Integrin-mediated dendrite branch maintenance requires Abelson (Abl) family kinases.** *J Neurosci* 2005, **25:**6105-6118.

3. Jones SB, Lu HY, Lu Q: **Abl tyrosine kinase promotes dendrogenesis by inducing actin cytoskeletal rearrangements in cooperation with Rho family small GTPases in hippocampal neurons.** *J Neurosci* 2004, **24:**8510-8521.

4. Allen KM, Walsh CA: **Genes that regulate neuronal migration in the cerebral cortex.** *Epilepsy Res* 1999, **36:**143-154.

5. Schmidt MH, Chen B, Randazzo LM, Bogler O:  **SETA/CIN85/Ruk and its binding partner AIP1 associate with diverse cytoskeletal elements, including FAKs, and modulate cell adhesion.**  *J Cell Sci* 2003, **116:**2845-2855.

6. Betz R, Sandhoff K, Fischer KD, Echten-Deckert G: **Detection and identification of Vav1 protein in primary cultured murine cerebellar neurons and in neuroblastoma cells (SH-SY5Y and Neuro-2a).** *Neurosci Lett* 2003, **339:**37-40.

7. Aoki K, Nakamura T, Fujikawa K, Matsuda M:  **Local phosphatidylinositol 3,4,5-trisphosphate accumulation recruits Vav2 and Vav3 to activate Rac1/Cdc42 and initiate neurite outgrowth in nerve growth factor-stimulated PC12 cells.** *Mol Biol Cell* 2005, **16:**2207-2217.

8. Mongiovi AM, Romano PR, Panni S, Mendoza M, Wong WT, Musacchio A, Cesareni G, Di Fiore PP: **A novel peptide-SH3 interaction.** *EMBO J* 1999, **18:**5300-5309.

9. Offenhauser N, Borgonovo A, Disanza A, Romano P, Ponzanelli I, Iannolo G, Di Fiore PP, Scita G: **The eps8 family of proteins links growth factor stimulation to actin reorganization generating functional redundancy in the Ras/Rac pathway.** *Mol Biol Cell* 2004, **15:**91-98.

10. Ieraci A, Forni PE, Ponzetto C: **Viable hypomorphic signaling mutant of the Met receptor reveals a role for hepatocyte growth factor in postnatal cerebellar development.** *Proc Natl Acad Sci U S A* 2002, **99:**15200-15205.

11. Huang J, Sakai R, Furuichi T: **The docking protein Cas links tyrosine phosphorylation signaling to elongation of cerebellar granule cell axons.** *Mol Biol Cell* 2006, **17:**3187-3196.

12. Cardoso C, Leventer RJ, Ward HL, Toyo-oka K, Chung J, Gross A, Martin CL, Allanson J, Pilz DT, Olney AH et al.: **Refinement of a 400-kb critical region allows genotypic differentiation between isolated lissencephaly, Miller-Dieker syndrome, and other phenotypes secondary to deletions of 17p13.3.** *Am J Hum Genet* 2003, **72:**918-930.

13. Smith CI, Islam TC, Mattsson PT, Mohamed AJ, Nore BF, Vihinen M: **The Tec family of cytoplasmic tyrosine kinases: mammalian Btk, Bmx, Itk, Tec, Txk and homologs in other species.** *BioEssays* 2001, **23:**436-446.

14. Lin J, Liu J, Wang Y, Zhu J, Zhou K, Smith N, Zhan X: **Differential regulation of cortactin and N-WASP-mediated actin polymerization by missing in metastasis (MIM) protein.** *Oncogene* 2005, **24:**2059-2066.

15. Hering H, Sheng M: **Activity-dependent redistribution and essential role of cortactin in dendritic spine morphogenesis.** *J Neurosci* 2003, **23:**11759-11769.

16. Hirokawa N, Takemura R: **Molecular motors and mechanisms of directional transport in neurons.** *Nat Rev Neurosci* 2005, **6:**201-214.

17. Li W, Fan J, Woodley DT: **Nck/Dock: an adapter between cell surface receptors and the actin cytoskeleton.** *Oncogene* 2001, **20:**6403-6417.

18. Wang YT, Linden DJ: **Expression of cerebellar long-term depression requires postsynaptic clathrin-mediated endocytosis.** *Neuron* 2000, **25:**635-647.

19. Kuo G, Arnaud L, Kronstad-O'Brien P, Cooper JA: **Absence of Fyn and Src causes a reeler-like phenotype.** *J Neurosci* 2005, **25:**8578-8586.

20. Nishio H, Matsui K, Tsuji H, Tamura A, Suzuki K: **Possible involvement of Fyn kinase in ethanol-stimulated Cas tyrosine phosphorylation in rat cerebellum and cerebral cortex.** *J Neurochem* 2001, **76:**1073-1079.

21. Cheng Q, Yeh HH: **PLCgamma signaling underlies BDNF potentiation of Purkinje cell responses to GABA.** *J Neurosci Res* 2005, **79:**616-627.

22. Disanza A, Mantoani S, Hertzog M, Gerboth S, Frittoli E, Steffen A, Berhoerster K, Kreienkamp HJ, Milanesi F, Fiore PP et al.: **Regulation of cell shape by Cdc42 is mediated by the synergic actin-bundling activity of the Eps8-IRSp53 complex.** *Nat Cell Biol* 2006, **8:**1337-1347.

23. Whitmarsh AJ, Kuan CY, Kennedy NJ, Kelkar N, Haydar TF, Mordes JP, Appel M, Rossini AA, Jones SN, Flavell RA et al.: **Requirement of the JIP1 scaffold protein for stress-induced JNK activation.** *Genes Dev* 2001, **15:**2421-2432.

24. Ha HY, Cho IH, Lee KW, Lee KW, Song JY, Kim KS, Yu YM, Lee JK, Song JS, Yang SD et al.: **The axon guidance defect of the telencephalic commissures of the JSAP1-deficient brain was partially rescued by the transgenic expression of JIP1.** *Dev Biol* 2005, **277:**184-199.

25. Taru H, Kirino Y, Suzuki T: **Differential roles of JIP scaffold proteins in the modulation of amyloid precursor protein metabolism.** *J Biol Chem* 2002, **277:**27567-27574.

26. Chen S, Ren YQ, Hillman DE: **Transient expression of lyn gene in Purkinje cells during cerebellar development.** *Brain Res Dev Brain Res* 1996, **92:**140-146.

27. Hayashi T, Umemori H, Mishina M, Yamamoto T: **The AMPA receptor interacts with and signals through the protein tyrosine kinase Lyn.** *Nature* 1999, **397:**72-76.

28. Sakakibara A, Horwitz AF: **Mechanism of polarized protrusion formation on neuronal precursors migrating in the developing chicken cerebellum.** *J Cell Sci* 2006, **119:**3583-3592.

29. Fukudome Y, Tabata T, Miyoshi T, Haruki S, Araishi K, Sawada S, Kano M: **Insulin-like growth factor-I as a promoting factor for cerebellar Purkinje cell development.** *Eur J Neurosci* 2003, **17:**2006-2016.

30. Huh GY, Glantz SB, Je S, Morrow JS, Kim JH: **Calpain proteolysis of alpha II-spectrin in the normal adult human brain.** *Neurosci Lett* 2001, **316:**41-44.

31. Sudol M, Kuo CF, Shigemitsu L, Alvarez-Buylla A: **Expression of the yes proto-oncogene in cerebellar purkinje cells.** *Mol Cell Biol* 1989, **9:** 4545-4549.

32. Omri B, Crisanti P, Marty MC, Alliot F, Fagard R, Molina T, Pessac B: **The Lck tyrosine kinase is expressed in brain neurons.** *J Neurochem* 1996, **67:**1360-1364.

33. Muraille E, Dassesse D, Vanderwinden JM, Cremer H, Rogister B, Erneux C, Schiffmann SN: **The SH2 domain-containing 5-phosphatase SHIP2 is expressed in the germinal layers of embryo and adult mouse brain: increased expression in N-CAM-deficient mice.** *Neuroscience* 2001, **105:**1019-1030.

34. Liu BA, Jablonowski K, Raina M, Arce M, Pawson T, Nash PD: **The human and mouse complement of SH2 domain proteins-establishing the boundaries of phosphotyrosine signaling.** *Mol Cell* 2006, **22:**851-868.

35. Ponti G, Conti L, Cataudella T, Zuccato C, Magrassi L, Rossi F, Bonfanti L, Cattaneo E: **Comparative expression profiles of ShcB and ShcC phosphotyrosine adapter molecules in the adult brain.** *Neuroscience* 2005, **133:**105-115.
