# Supplementary material for: Developmental expression and differentiation-related neuron-specific splicing of metastasis suppressor 1 (Mtss1) in normal and transformed cerebellar cells
Source: BMC Dev Biol. 2007 Oct 9;7:111. doi: 10.1186/1471-213X-7-111 (PMC2194783; doi:10.1186/1471-213X-7-111)
Supplement: Additional file 3 — SH2-domain binding motifs found in Mtss1. List of potential SH2-domain binding motifs identified in Mtss1. [file 1471-213X-7-111-S3.doc]

**Supplemental table 3**

SH2-domain binding motifs found in Mtss1

| **Motif center** | **Sequence** | **score** | **percentile** | **domain** | **Allen** | **BGEM p7** | **BGEM Ad** | **References** | **selected functions** |
| --- | --- | --- | --- | --- | --- | --- | --- | --- | --- |
| Y260  Y422 | GSDYSWSYQTPPSSP  DWAKPGPYDQPLVNT | 0.2712  0.4966 | 0.005  1.13 | Abl | Pj+; gc(+) | egl, igl, Pj? | gc, Pc? | [1-4] | neuronal migration, actin rearrangement causative for dendritogenesis; postsynaptic role in synaptogenesis; dendrite maintenance |
| Y260  Y422 | GSDYSWSYQTPPSSP  DWAKPGPYDQPLVNT | 0.3404  0.4614 | 0.03  0.451 | Nck | see table 2, data for Nck1 and Nck2 | | | | |
| Y177  Y260 | LQDVNDKYLLLEETE  GSDYSWSYQTPPSSP | 0.3668  0.4991 | 0.452  2.206 | PLCgamma N-terminal  PLCgamma C-terminal | Pj+; gcl(+) | no data | no data | [21] | mediates BDNF potentiation of GABA signaling |
| Y260 | GSDYSWSYQTPPSSP | 0.4251 | 0.13 | Itk | Pj very weak | no data | no data | [13] | no documented cns action |
| Y260  Y397  Y547 | GSDYSWSYQTPPSSP  HLPDYAHYYTIGPGM  GPVSDYDYFSVSGDQ | 0.4571  0.5271  0.5303 | 1.435  4.539  4.763 | Lck  Lck  Lck | cerebell neg | egl(+), igl(+) | not informative | [32] | expressed in adult gc and PJs, dendritic localization |
| Y397 | HLPDYAHYYTIGPGM | 0.5053 | 2.940 | Src | Pj++ | egl+; igl+; Pj? | Pcl, igl(+) | [19] |  |
| Y177 | LQDVNDKYLLLEETE | 0.5181 | 3.352 | INPP5D | no exp in cerebell. | no data | no data | [33] | immuologic function; no neural function known |
| Y260  Y397 | GSDYSWSYQTPPSSP  HLPDYAHYYTIGPGM | 0.5356  0.5563 | 2.384  3.296 | Fyn  Fyn | Pcl+ | egl+, igl+ | not informative | [19,20] | reelin pathway; ethanol damage |
| Y260 | GSDYSWSYQTPPSSP | 0.5549 | 2.795 | Shc | Pj neg | egl++, Igl(+) Pj? | igl+ |  | reduced proliferation, enhanced apoptosis; |
| *Y260* | *GSDYSWSYQTPPSSP* | *0.5549* | *2.795* | shc2 | Pj+ | egl++, igl(+) | igl neg | [34,35] |  |
| *Y260* | *GSDYSWSYQTPPSSP* | *0.5549* | *2.795* | shc3 | Pj+ | no data | no data | [34,35] |  |

Sequence numbering gives the phosphorylation site defining the binding site, and refer to the sequence of Mtss1 containing exon 12, the N-terminally extended version of exon 14, and the complete (i.e., unspliced) variant of exon 15 (cf Fig 2). Prediction scores (best is 0, worst is +∞) and percentiles are those reported by Scansite. For references, see legend to supplemental table 2
