# Supplementary material for: Developmental expression and differentiation-related neuron-specific splicing of metastasis suppressor 1 (Mtss1) in normal and transformed cerebellar cells
Source: BMC Dev Biol. 2007 Oct 9;7:111. doi: 10.1186/1471-213X-7-111 (PMC2194783; doi:10.1186/1471-213X-7-111)
Supplement: Additional file 4 — Binding motifs for 14-3-3-, FHA- and WW class IV-domain proteins in Mtss1. Potential binding motifs identified in Mtss1 for proteins with 14-3-3-, FHA- or WW class IV domains. [file 1471-213X-7-111-S4.doc]

Supplemental table 4

Binding motifs for 14-3-3-, FHA- and WW class IV-domain proteins in Mtss1.

| **14-3-3- binding motifs (ScanSite predictions)** | | | |
| --- | --- | --- | --- |
| **Motif center** | **sequence** | **Score** | **Percentile** |
| T466 | AQRPRSMTVSAATRP | 0.3550 | 0.229 |
| S464 | EEAQRPRSMTVSAAT | 0.4105 | 0.456 |
| S307 | SSHYRYRSSNLAQQA | 0.4455 | 0.697 |
| S320 | QAPVRLSSVSSHDSG | 0.5055 | 1.390 |
| T566 | QEFDKSSTIPRNSDI | 0.5093 | 1.445 |
| T641 | VIPVKTPTVPDLPGV | 0.5201 | 1.610 |
|  | | | |
| **FHA-binding motifs (ELM predictions)** | | | |
| **Motif start** | **sequence** |  |  |
| 47 | TVVA |  |  |
| 72 | TREI |  |  |
| 144 | TLKL |  |  |
| (ex12a)(380) | TMGA |  |  |
| 466 (441) | TVSA |  |  |
| 484 (459) | TLAL |  |  |
| 589 (564) | TAGL |  |  |
| 622 (597) | TIGA |  |  |
| 632 (607) | TPVI |  |  |
|  | | | |
| **WW class IV-binding motifs (ELM predictions)** | | | |
| **Motif start** | **sequence** |  |  |
| 259-264 | SYQTPP |  |  |
| 263-268 | PPSSPS |  |  |
| 295-300 | HSHSPS |  |  |
| 335-340 | QSKSPS |  |  |
| 337-342 | KSPSPM |  |  |
| (ex 12a) (368-373) | ECSSPT |  |  |
| 512-517 (487-492) | QTTTPC |  |  |
| 599-604) 574-579 | AMVTPG |  |  |
| 608-613 (583-588) | IRRTPS |  |  |
| 629-634 (604-609) | PIKTPV |  |  |
| 636-641 (611-616) | PVKTPT |  |  |
| 648-653 (623-628) | VLPSPP |  |  |
| 660-665 (635-640) | GEHSPE |  |  |
| 663-668 (638-643) | SPESPS |  |  |
| 724-729 (699-704) | ATVSPG |  |  |
| 732-742 (712-717) | ADLSPR |  |  |

Sequence numbering is as for table 2. In addition, numbers in parenthesis

give positions in the exon 12a variant of Mtss1. Scores and percentiles for

FHA binding motifs are from Scansite (cf legend to supplemental table 2).
